# Prevalence and characteristics of factitious hypoglycaemia in non‐diabetic patients in a department of endocrinology

**DOI:** 10.1002/edm2.375

**Published:** 2022-09-18

**Authors:** Ibtissem Oueslati, Amani Terzi, Meriem Yazidi, Elyes Kamoun, Melika Chihaoui

**Affiliations:** ^1^ Department of Endocrinology, La Rabta University Hospital, Faculty of Medicine University of Tunis‐El Manar Tunis Tunisia

**Keywords:** factitious hypoglycaemia, hypoglycaemia in non‐diabetic patients, insulin, insulin secretagogues medications, psychiatric care

## Abstract

**Introduction:**

Factitious hypoglycaemia is defined as the surreptitious use of insulin or oral hypoglycaemic agents to deliberately induce self‐harm. It represents a challenging diagnosis and misdiagnosis is associated with significant morbidity and mortality.

The aim of this study was to assess the prevalence and the associated factors of factitious hypoglycaemia in non‐diabetic patients.

**Methods:**

This was a single‐centre, retrospective study including 70 non‐diabetic patients who were admitted for the investigation of hypoglycaemia. All patients fulfilled the Whipple triad. Epidemiological parameters, medical history, clinical and paraclinical data and the aetiology of hypoglycaemia were collected from medical records.

**Results:**

The diagnosis of factitious hypoglycaemia was held in 11 patients (9 women and 2 men) corresponding to a prevalence of 16%. It was secondary to intentional insulin use in six patients and the ingestion of glibenclamide in five patients. The median age of the patients was 28 years (interquartile range: 21–43). Two patients with factitious hypoglycaemia had a personal history of psychiatric disorders. The other causes of hypoglycaemia were adrenal insufficiency (34%), prediabetes (24%), insulinoma (6%), iatrogenic hypoglycaemia (10%), criminal hypoglycaemia (1%) and alcohol intoxication (2%). Age ≤ 35 years (Odds Ratio = 5.6, *p* = .017), family history of diabetes mellitus (Odds Ratio = 1.29, *p* = .015), attention disorders during hypoglycaemia (Odds Ratio = 12.5, *p* = .017) and fasting glucose level <0.7 g/L (Odds Ratio = 5.75, *p* = .017) were positively associated with factitious hypoglycaemia.

**Conclusion:**

Factors significantly associated with factitious hypoglycaemia were young age, family history of diabetes and a low fasting glucose level.

## Graphical abstract

According to our data, the significant differences between the reference population (other causes of hypoglycemia) and factitious hypoglycemia were age, family history of diabetes and a lower fasting glucose levels. A psychiatric referral is warranted to provide the appropriate care for patients.

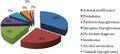

## 1 INTRODUCTION

Hypoglycaemia in non‐diabetic patients is a rare metabolic emergency. It presents with variable and non‐specific symptoms including adrenergic and/or neuroglycopenic signs. The diagnosis of hypoglycaemia is challenging. It requires the fulfilment of the Whipple triad which consists of the presence of clinical symptoms, a concomitant blood glucose level lower than 2.75 mmol/L (50 mg/dl) and the resolution of symptoms after glucose administration and correction of hypoglycaemia.^1^

Hypoglycaemia in non‐diabetic adults may be due to multiple aetiologies such as primary and secondary adrenal insufficiency, insulinomas, non‐insulinoma pancreatogenous hypoglycaemia syndrome, non‐islet cell tumour hypoglycaemia, autoimmune syndrome, critical illness and functional hypoglycaemia.^2^ Factitious hypoglycaemia is defined by an intentional insulin use or ingestion of oral hypoglycaemic agents to induce self‐harm, seek attention and gain care. It is a rare disorder commonly associated with significant morbidity and mortality. It is usually denied during the history approach leading to a challenging diagnosis and a life‐threatening condition if missed.^3^

The aim of this study was to assess the prevalence and the characteristics of factitious hypoglycaemia in non‐diabetic patients in a department of endocrinology.

## 2 METHODS

This was a single‐centre, retrospective study including 70 non‐diabetic patients who were admitted to the department of endocrinology of La Rabta university hospital in Tunis, between 2004 and 2020 for the investigation of hypoglycaemia. All enrolled patients fulfilled the Whipple triad. Exclusion criteria were as follows: Age ≤ 14 years and pregnancy. Age, gender, family history of diabetes, personal history of psychiatric disorders, occupation, socioeconomic condition, smoking, alcohol consumption, hypoglycaemia symptoms, the frequency of hypoglycaemia, body weight, body mass index, fasting blood glucose, blood glucose level during hypoglycaemia, insulin and C‐peptide levels during hypoglycaemia and the aetiology of hypoglycaemia were collected from medical records. Fasting blood glucose was measured using the hexokinase/G6PDH method. Insulin and C‐peptide levels were measured using an electro‐chemiluminescence immunoassay.

### 2.1 Statistical analysis

Statistical analysis was performed using the SPSS software package version 22. Quantitative variables were expressed as mean ± standard deviation (SD) or as the median and interquartile range (IQR). Qualitative variables were expressed in percentages. The nonparametric Mann–Whitney test was used to compare quantitative variables and the Fisher exact test to compare qualitative variables. *p* values < .05 were considered to indicate statistical significance.

## 3 RESULTS

A total of 70 non‐diabetic patients (48 women and 22 men) with hypoglycaemia were enrolled in the study. Their mean age was 44.7 ± 18.6 years.

**FIGURE 1:**
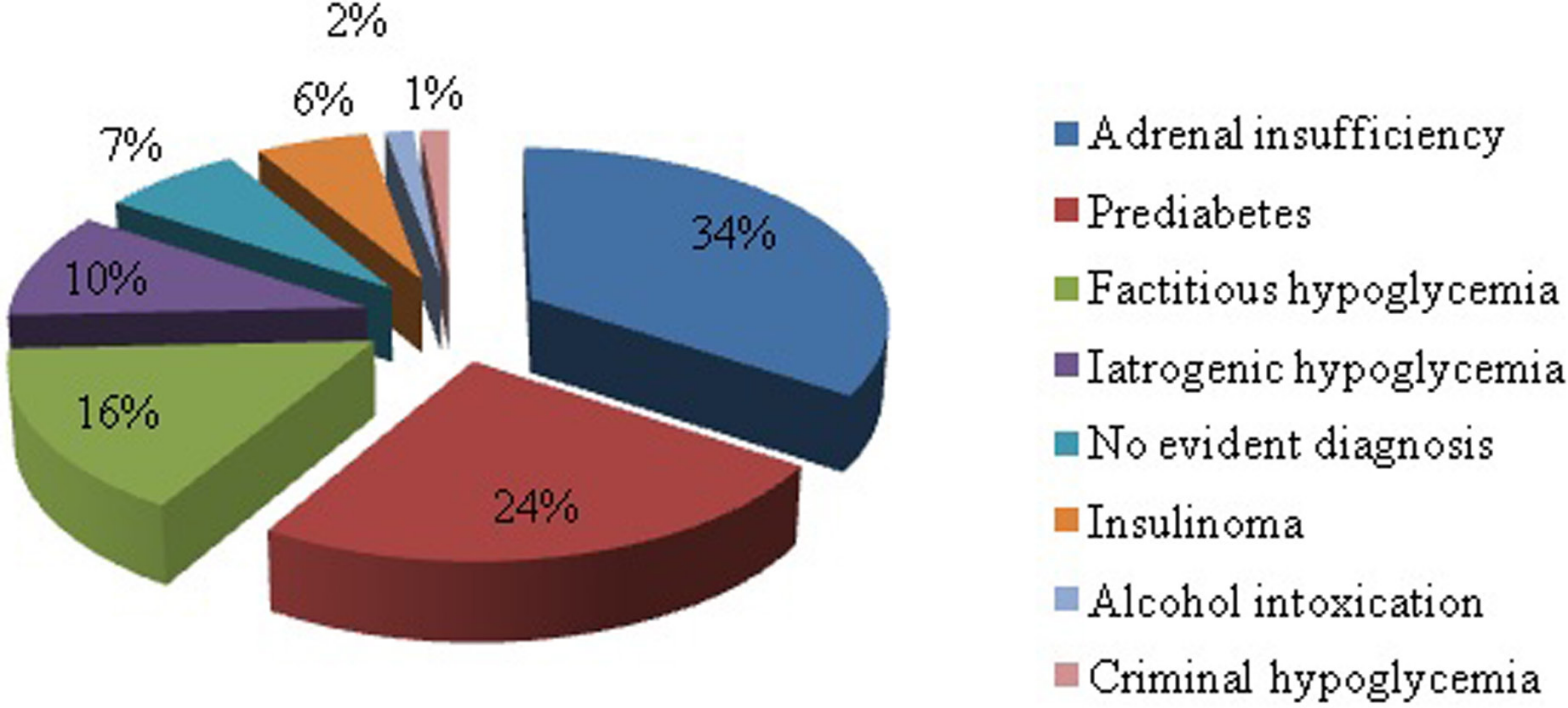
Aetiologies of hypoglycaemia in the study population

Figure 1 represents the different aetiologies of hypoglycaemia. The most frequent causes of hypoglycaemia were adrenal insufficiency (34%) and pre‐diabetes (24%). The diagnosis of criminal hypoglycaemia, defined as third‐party induced hypoglycaemia with a will of murder, was established in one case (1%). In five patients (7%), all the investigations enrolled were negative and the aetiology of hypoglycaemia was undetermined.

Factitious hypoglycaemia was diagnosed in 11 patients corresponding to a prevalence of 16%. It was secondary to intentional rapid‐acting insulin use via subcutaneous injection in six patients and the ingestion of glibenclamide in five patients.

Table 1 represents the comparison of epidemiological, clinical and biological data between patients with factitious hypoglycaemia and those with other causes of hypoglycaemia.

**TABLE 1:** Epidemiological, clinical and biological characteristics of patients with factitious hypoglycaemia and those with other causes of hypoglycaemia

		Factitious hypoglycaemia (*n* = 11)	other causes of hypoglycaemia (*n* = 59)	*p*	OR	CI‐95%
Age, median (IQR) (years)		28 (21–43)	45 (32–64)	**.005**		
Age ≤ 35 years (%)		73	32	**.017**	5.61	1.33–23.57
Women (%)		91	64	.154	‐	‐
Smoking (%)		27	30	1		
Alcohol consumption (%)		0	10	.58		
Low socioeconomic condition (%)		44	31	.456		
Unemployed (%)		44	61	.469		
Medical profession (%)		0	3	.708		
Family history of diabetes, (%)		100	64	**.015**	1.29	1.11–1.51
Personal history of psychiatric disorders, (%)^a^		18	8	.302		
Hypoglycaemia symptoms, (%)	Asthenia	90	86	1		
	Tremor	90	72	.423		
	Sweating	100	89	.579		
	Palpitations	91	85	1		
	Hunger	75	50	.603		
	Anxiety	50	54	1		
	Dizziness	67	56	.685		
	Visual flow	62	44	.451		
	Attention disorder	83	29	**.017**	12.5	1.31–118.47
	Behaviour disorder	50	27	.569		
	Slurred speech	50	15	.145		
	Disorientation	67	26	.067		
	Seizure	57	29	.201		
	Coma	67	33	.073		
Frequency of hypoglycaemia/week		3	3	.801		
Body weight, median (IQR) (Kg)		68.5 (56–80.5)	71 (59–82)	.938		
BMI, median (IQR) (kg/m^2^)		24.8 (20.2–30.3)	25.7 (23.0–29.0)	.634		
FG level, median (IQR) (mmol/L)		3.63 (2.25–5.11)	4.67 (4.12–5.11)	.168		
FG level <3.85 mmol/L, (%)		60	21	**.017**	5.75	1.39–23.68

^a^ Depression in the two patients with suicide attempts in one case.

Abbreviations: BMI, body mass index; CI, confidence interval; FG, fasting glucose; IQR, interquartile range; OR, Odds Ratio.

The median level of glycemia in patients with factitious hypoglycaemia due to the ingestion of glibenclamide was 2.14 mmol/L (IQR: 1.81–2.58). During hypoglycaemia, their median insulin level was 20.3 mUI/L (IQR: 19–20.3) and their C‐peptide level was 2.79 μg/L (IQR:0.79–2.79). In patients with factitious hypoglycaemia due to self‐insulin injection, the median level of glycemia was 1.98 mmol/L (IQR: 1.26–2.14). Their median insulin level during hypoglycaemia was 58 mUI/L (IQR: 33.7–390.5) and their C‐peptide level was 0.21 μg/L (IQR: 0.06–0.28).

When confronted with evidence about factitious hypoglycaemia, all patients admitted the intentional insulin injecting or glibenclamide ingestion. They were referred to the psychiatry department. However, they were all lost to follow‐up.

## 4 DISCUSSION

Hypoglycaemia is uncommon in non‐diabetic patients. Nirantharakumar et al.^4^ reported that with a cut‐off value of 2.7 mmol/L, 36 episodes of non‐diabetic hypoglycaemia per 10,000 admissions occurred per year outside the critical care setting. Factitious hypoglycaemia is a serious disease given the significant morbidity, mortality and demonstrated loss to follow‐up. It is one of the most challenging disorders in the medical experience that represents a difficult diagnosis due to the complication of capturing events, the lack of forthcoming and the incident of false medical history. It is rare and under‐diagnosed. The overall prevalence of factitious disorders is estimated to be 0.5%–2%.^5^, ^6^, ^7^ Yates et al.,^8^ in a systematic review of 455 cases with factitious disorders in the professional literature, found 31 cases with self‐induced hypoglycaemia. In the retrospective study of Bérar et al.,^9^ 49 patients with factitious disorders were included among them factitious hypoglycaemia was identified in two patients (4%). According to Nirantharakumar et al.,^9^ the prevalence of factitious hypoglycaemia was 10.8% in non‐diabetic patients with hypoglycaemia. In many studies, factitious hypoglycaemia was reported as case reports.^10^, ^11^, ^12^, ^13^ Our study provided an unprecedented opportunity to estimate the prevalence of factitious hypoglycaemia (16% in this sample) since the particularly notable paucity of reports outlining this setting. However, this prevalence may be underestimated since the aetiology of hypoglycaemia was undetermined in 7% of our patients.

The age of patients with factitious hypoglycaemia in our sample corroborates the results of previous observations.^8^, ^11^, ^12^ Whilst it can be seen in broadly different ages ranging from 8^14^ to 82 years old,^15^ cases with factitious hypoglycaemia seem to be occurring more frequently in early adult life. In this regard, patients with deliberate attempts to induce low serum glucose levels are most commonly middle‐aged. Similarly, we conclude to a female predominance in our study that asserts the evidence in the existing literature that patients with factitious hypoglycaemia are more likely female.^11^ The reasons behind the higher occurrence of factitious disorder in women than men could not be discerned. It may be due to the higher rate of psychiatric illness in women in general.^11^, ^16^

In this study, all patients with self‐harm intent had a relative with diabetes mellitus which explains the access to insulin or insulin‐secretagogues. Similar findings were reported previously.^12^, ^17^, ^18^ It is of note that these patients were familiar with the hypoglycaemic effects of insulin or the oral antidiabetic agents.^19^

Concerning occupation, none of the patients in our study did have a medical profession. However, numerous papers report that factitious disorders are more frequent in medical professionals.^11^ This may be explained by the ease of access to drugs and medical knowledge.

In our study, two patients with factitious hypoglycaemia had a history of depression with suicide attempts in one case. According to many authors, higher rates of co‐existing psychiatric illnesses were detected in patients with factitious hypoglycaemia.^8^, ^11^, ^12^, ^20^ A considerable number of reports assert that comorbid psychiatric conditions such as personality disorders, mood disturbances, anxiety, depression, substance abuse, childhood offence and suicidal thoughts were strongly associated with factitious hypoglycaemia.^10^, ^21^ The reasons behind this self‐destructive behaviour are mostly the need to escape ordinary life constraints, seek medical attention and gain empathy.

It is noted that patients with factitious hypoglycaemia may falsify medical history, mimic signs, tamper with features, interfere with diagnostic investigations to induce an illness and seek medical care through unnecessary tests and repeated hospital admissions. Thus, the clinical presentation of self‐induced hypoglycaemia is highly variable. Patients may present with a spectrum of symptoms including asthenia, tremor, sweating, palpitations, hunger, dizziness, blurred vision, slurred speech and attention disorders or difficulties in concentration.^10^ The latter was significantly more frequent in patients with factitious hypoglycaemia than patients with other causes of hypoglycaemia in our study. Patients with factitious hypoglycaemia are more likely to undergo severe complications of hypoglycaemia such as disorientation, seizures, brain damage, irreversible neurological sequelae, coma and death.^10^, ^11^, ^17^, ^20^, ^22^

Factitious hypoglycaemia is a challenging diagnosis. Measuring insulin, proinsulin and C‐peptide levels during a hypoglycaemic event help differentiate it from other causes of hypoglycaemia. In the case of exogenous intentional human insulin use, the plasma insulin level is elevated while the proinsulin and C‐peptide levels are suppressed. However, in the case of insulin analogue injection, the diagnosis may be more difficult since most commercial insulin assays can detect only human insulin and fail to detect insulin analogues due to low cross‐reactivity.^13^, ^23^ The detection power of a test for insulin analogues depends on both the type of platform and the complexity of the analogue.^13^ On the contrary, in the case of auto‐ingestion of sulfonylureas that stimulate endogenous insulin secretion, insulin, proinsulin and C‐peptide levels are elevated mimicking thereby an insulinoma. The drug detection in the blood or urine adjusts the diagnosis.^24^ Clinical suspicion remains thus the fundamental key to establish the diagnosis considering the shortage of any definitive laboratory findings.^13^ In some cases, as in our study, patients may admit to the intentional use of hypoglycaemic drugs.

Among the aetiologies of hypoglycaemia in our study population, 7% of cases were undetermined. Factitious hypoglycaemia may be under‐diagnosed in these cases due to the shortage of diagnostic tools in terms of detecting insulin analogues or compounds such as glimepiride and repaglinide as well as glibenclamide.

The immediate treatment of factitious hypoglycaemia which is a life‐threatening emergency requires the rapid administration of glucose to resurrect a normal blood level. Further treatment warrants a multidisciplinary approach that involves the general practitioner, the psychiatrist and the social worker.^12^ Indeed, psychiatric referral with constructive confrontation and appropriate follow‐up strategies are paramount to embrace the patient's situation, gain compliance and encourage a supportive process to achieve proper mental health. Patel et al.^10^ assert that psychotherapy is the paradigmatic key to stabilizing the self‐destructive behaviour in patients with factitious hypoglycaemia. In addition, a social support network in the long‐term management of this disorder is important.^11^ Nevertheless, most patients with factitious hypoglycaemia in our study are lost to follow‐up after confrontation and establishment of the diagnosis while reports highlighting long‐term follow‐up results of patients with factitious hypoglycaemia disclose gloomy prognoses with damaging consequences and poor adherence to treatment.^10^, ^11^

## 5 CONCLUSION

Factitious hypoglycaemia in non‐diabetic patients due to self‐administration of insulin or oral antidiabetic drugs represents a diagnostic challenge that continues nowadays to intrigue clinicians. According to our data, the significant differences between the reference population (other causes of hypoglycaemia) and factitious hypoglycaemia were age, family history of diabetes and a lower fasting glucose levels. A stepwise approach and a psychiatric assessment are necessary to provide patients with the appropriate guidance and to ensure a supportive follow‐up.

## AUTHOR CONTRIBUTIONS

**Amani Terzi:** Data curation (supporting); investigation (supporting); methodology (supporting); resources (supporting); writing – original draft (supporting); writing – review and editing (supporting). **Elyes Kamoun:** Conceptualization (supporting); data curation (supporting); methodology (supporting); resources (supporting); writing – review and editing (supporting). **Melika Chihaoui:** Conceptualization (supporting); data curation (supporting); investigation (supporting); methodology (equal); project administration (supporting); resources (supporting); supervision (equal); validation (equal); visualization (supporting); writing – review and editing (equal). **Meriem Yazidi:** Conceptualization (supporting); data curation (supporting); investigation (supporting); methodology (supporting); validation (supporting); writing – review and editing (supporting). **Ibtissem Oueslati:** Conceptualization (equal); data curation (equal); formal analysis (equal); funding acquisition (equal); investigation (equal); methodology (equal); project administration (equal); resources (equal); software (equal); supervision (equal); validation (equal); visualization (equal); writing – original draft (equal); writing – review and editing (equal).

## FUNDING INFORMATION

This work did not receive any specific grant from funding agencies in the public, commercial or not‐for‐profit sectors.

## CONFLICT OF INTEREST

The authors declare that they have no conflict of interest.

### ETHICS STATEMENT

This study has been approved by the appropriate ethics committee and has therefore been performed in accordance with the ethical standards laid down in the 1964 Declaration of Helsinki and its later amendments.

## Data Availability

The data used to support the findings of this study are available from the corresponding author upon reasonable request.
